# Transnasal humidified rapid insufflation ventilatory exchange for pre-oxygenation and apnoeic oxygenation during rapid sequence induction

**DOI:** 10.1186/cc14288

**Published:** 2015-03-16

**Authors:** M Mariyaselvam, D Stolady, G Wijewardena, M Blunt, P Young

**Affiliations:** 1 Queen Elizabeth Hospital, King's Lynn, UK

## Introduction

Rapid sequence induction (RSI) in the ICU, emergency department (ED) and operating room (OR) carries the risk of hypoxemia if laryngoscopy is prolonged especially in high-risk patients. Bag and mask pre-oxygenation is normally used to extend the apnoea time; however, arterial desaturation may still rapidly occur. Transnasal humidified rapid insufflation ventilatory exchange (THRIVE) is a new technique that provides modest CPAP during pre-oxygenation and crucially also continuous oxygenation of the pharyngeal space throughout the apnoeic period. In elective surgery, THRIVE provides apnoea times as long as 60 minutes due to apnoeic oxygenation [1]. We report the first implementation of THRIVE with emergency patients into the ICU, ED and OR.

## Methods

Following training a THRIVE system was installed in each location either as a fixed system on the anaesthetic machine (OR) or a mobile solution on a wheeled stand (ICU, ER). This was a simplified Optiflow system (Fisher and Paykel, New Zealand) consisting of a high-flow rotameter, a reusable humidifier, a reusable circuit and a disposable nasal interface. Anaesthetists of all grades were encouraged to use THRIVE (60 l/minute) prior to and during all high-risk intubations. Prospective data of pre and post intubation SpO_2_ and time to intubate were collected. Anaesthetists were interviewed on acceptability of the technique.

## Results

There were 62 RSI intubations using THRIVE (ICU and ED = 30; OR = 33). Difficult airway equipment used in 36 cases (videolaryngoscopy in 23). Mean apnoea time was 118 seconds (30 to 480 seconds), with a median SpO_2_ fall of 1% (0 to 33%). There was no correlation between arterial desaturation and apnoeic time. OR cases had a mean apnoea of 113 seconds with a median SpO_2_ fall of 0% (0 to 13%). ICU and ED cases had a mean apnoea time of 119 seconds and median SpO_2_ fall of 1% (0 to 33%). THRIVE was universally readily accepted. Reasons cited included: simplification of pre-oxygenation (hands free) and increased confidence. Six outlying arterial desaturation events suggested poor airway maintenance at induction or use in particularly high-risk patients. Many anaesthetists reinstituted THRIVE following extubation in selected patients (for example, obesity). No complications occurred during implementation.

## Conclusion

We conclude that THRIVE provides a convenient, safe and easy to implement technique for pre-oxygenation and apnoeic oxygenation during laryngoscopy.
